# Characteristics and predicting factors of Corona Virus Disease-2019 (COVID-19) among healthcare providers in a developing country

**DOI:** 10.1371/journal.pone.0245672

**Published:** 2021-01-20

**Authors:** Rehab H. El-Sokkary, Amani El-Kholy, Sally Mohy Eldin, Walaa Shawky Khater, Doaa Mostafa Gad, Shereen Bahgat, Essam Edin M. Negm, Jehan Ali El Kholy, Sherif Mowafy, Eman Mahmoud, Eman M. Mortada

**Affiliations:** 1 Medical Microbiology and Immunology Department, Faculty of Medicine, Zagazig University, Zagazig, Egypt; 2 Clinical Pathology Department, Faculty of Medicine, Cairo University, Cairo, Egypt; 3 Ministry of Health and Population, Cairo, Egypt; 4 Medical Microbiology and Immunology Department, Faculty of Medicine, Ain Shams University, Cairo, Egypt; 5 Chest Department, Faculty of Medicine, Zagazig University, Zagazig, Egypt; 6 Family Medicine Department, Faculty of Medicine, Zagazig University, Zagazig, Egypt; 7 Anesthesia & Surgical Intensive Care Department, Faculty of Medicine, Zagazig University, Zagazig, Egypt; 8 Anesthesia & Surgical Intensive Care Department, Faculty of Medicine, Cairo University, Cairo, Egypt; 9 Infection Prevention and Control Department, Dar Al Fouad Hospital, Nasr City, Cairo, Egypt; 10 Microbiology and Immunology Department, National Liver Institute, Menoufeya University, Shibin el Kom, Egypt; 11 Community, Occupational and Environmental Medicine Department, Faculty of Medicine, Zagazig University, Zagazig, Egypt; 12 Health Sciences Department, Health Sciences & Rehabilitation College, Princess Nourah Bint Abdulrahman University, Riyadh, Saudi Arabia; Federation University Australia, AUSTRALIA

## Abstract

A limited number of publications have identified risk factors for Corona Virus Disease 2019 (COVID-19) among Healthcare Providers (HCPs). We aimed to assess the clinical and epidemiological characteristics and the predicting factors related to COVID-19 among HCPs in Egypt. A comparative cross-sectional study was conducted among HCPs via an online questionnaire. Out of 440 responses, a total of 385 complete responses were analyzed. The responders’ mean age was 37.5±9.4 years, 215 (55.8%) of the participants were males. They included 77 (20%) confirmed COVID-19 cases; most of them had mild (58.6%) or moderate symptoms (30%), and (9.1%) were asymptomatic. Almost all sustained infection while on duty (97.4%). The sources of infection were either infected patients (39%), colleagues (22.1%), household contacts (5.2%) or uncertain (33.8%). The sources were symptomatic in only 62.3% of cases. Asymptomatic or pre-symptomatic sources accounted for 37.7% of the cases. Exposure occurred during healthcare provision in 66.3% of the cases. The presence of co-morbidities (OR = 2.53, CI 1.47–4.38, P = 0.001), working more than 8 hours per day in isolation hospital (OR = 3.09, CI 1.02–9.35, P = 0.046), training on hand hygiene (OR = 2.31, CI 1.05–5.08, P = 0.038) and adherence to IPC measures (OR = 2.11, CI 1.16–3.81, P = 0.014) were the significant predictors of COVID-19. In conclusion, COVID-19 occurred in 20% of responders. Silent spread from asymptomatic or presymptomatic patients, and infected colleagues in hospital settings is an alarming sign. Proactive infection prevention and control measures are highly encouraged on both strategic and operational levels. Reconsideration of surveillance strategy and work-related regulations in healthcare settings are warranted.

## Introduction

The novel enveloped RNA beta coronavirus, named “Severe Acute Respiratory Syndrome Coronavirus 2” (SARS-CoV-2), has caused a variety of clinical manifestations ranging from mild respiratory symptoms up to severe illness and death in China and other countries since December 2019 [1, 2]. The disease caused by the emerging virus was termed Corona Virus Disease 2019 (COVID -19) by the World Health Organization (WHO) [3]. On March 11, the WHO categorized the COVID-19 outbreak as a global pandemic [4], and subsequent worldwide aggressive actions have since been taken to mitigate the spread of the infection. Egypt publicized its first COVID-19 case on February 14, 2020 [5]. As of July 2^nd^, the Egyptian authorities announced a total of 69814 confirmed COVID-19 cases [6]. However, an underestimation of the total number of cases is anticipated, as clarified by the Egyptian minister of health [7].

More than 22,000 healthcare providers (HCPs) have already been infected worldwide [8]. This number probably under-represents the true number of COVID-19 HCPs cases due to absence of systematic reporting for such infections to the WHO. There are a limited number of publications and national situation reports that provide relevant information where infections among HCPs accounted for 4.4% and 11% in China and Italy respectively [8]. The Center for Disease Control and Prevention (CDC) reported that 19% of COVID-19 patients in the United States were HCPs [9]. In Egypt, no official estimates of the number/rate of SARS-CoV-2 infected HCPs exist so far.

HCPs being at high risk for acquiring infections during the epidemic chain is a critical issue due to their active role in controlling the situation. Worldwide, and particularly in low and middle-income countries with limited resources and understaffing in medical staff in many healthcare settings, it is fundamental to keep HCPs safe. Consequently, all possible actions must be considered to control the spread of the infection to them, first by identifying the risk factors related to infection and then taking appropriate measures to reduce these risks [10].

The complicated healthcare delivery system in Egypt [11], with many HCPs working on part-time basis in more than one medical setting, the limited availability of resources and understaffing in many of its hospitals would probably add to challenges of curbing the disease transmission among HCPs and the community. The survey demonstrates in details the characters and risk factors of COVID-19 among healthcare providers which, if considered in EGYPT or any other developing country of similar healthcare system and economic conditions, could give a hand to slow-down the global spread of disease. Researchers tried to address the knowledge, attitude of HCPs towards COVID-19 as well as the psychological impact [12–15]. Khalifa et al., underscore the urgent need to educate the health care workers in Egypt about how to protect themselves [16].

However, and to the best of our knowledge, no studies investigated COVID-19 infections among Egyptian HCPs so far, especially those pertaining to key risk determinants of infection.

To bridge the aforementioned gap, two research questions were put forward: what are the clinical and epidemiological characteristics, and what are the predicting factors of COVID-19 among HCPs in Egypt. Based on self-reported data, the study was conducted among Egyptian HCPs to assess the frequency, the characteristics and the predicting factors of COVID-19 among HCPs in Egypt.

## Methods

### Study setting and participants

This cross-sectional study was conducted in Egypt, a middle-income country in the northeast corner of Africa. The study is a questionnaire-based survey conducted among HCPs from mid-June to mid-July 2020.

HCPs who agreed to fill in the questionnaire were considered eligible for participation. Participants’ inclusion criteria included complete responses from HCPs working inside Egypt during COVID-19 pandemic. The questionnaire included screening questions that helped to disqualify participants who didn’t meet the inclusion criteria.

According to their responses, participants were classified into two groups:

Group I: comprised HCPs who did not have COVID-19.

Group II: comprised HCPs who had COVID-19. This group included only laboratory confirmed COVID-19 cases as defined by the WHO and CDC [17, 18], whereas probable cases were excluded from the study.

The study was conducted according to the international guidelines of Strengthening the Reporting for Observational Studies in Epidemiology; STROBE [19]. Based on 80% degree of precision at 95% confidence interval (CI), the estimated sample size (385) for the study was calculated using online sample size calculator. To avoid missed responses, a total of 440 responses were collected. A snowball and convenient sampling technique were used.

### Data collection tool

A self-administered questionnaire was developed with reference to previously published reports [20–23] and was made available in both English and Arabic languages. To ensure acceptability, clarity of the questions, and face validity, the survey questionnaire was pilot tested on 15 HCPs who were excluded from further study analysis. Modifications were made as deemed necessary to avoid any ambiguity and ensure user-friendliness of the survey. The final version of the survey could be completed in less than ten minutes (S1 File).

The questionnaire consisted of four sections. The first section covered demographic and workplace characteristics. The second section included questions addressing infection prevention and control (IPC) measures. The third section included questions measuring factors that might lead to acquired infection among HCPs while interacting with COVID-19 patients. They were subsequently classified as having high or low risk of exposure according to WHO guidance for risk of exposure [23]. The fourth part included questions assessing clinical and epidemiological characteristics of HCPs who acquired COVID-19. Guided by the national management protocol [24], they were classified by their treating physicians and laboratory work up as having mild, moderate and severe symptoms.

### Data collection procedure

The strategy of online distribution of the survey involved emails and social media platforms, including WhatsApp, Facebook, Twitter and Telegram. We targeted professional groups that gather Egyptian HCPs from different disciplines and specialties. Participants were encouraged to share the survey with their colleagues to ensure maximal participation. Detailed instructions about the research objectives were illustrated in the participation form.

### Ethical considerations

Ethical approval was obtained from Zagazig University Institutional Review Board (IRB) (ZU-IRB#: 6207-23-6-2020). Participation was voluntary, anonymity was ensured as no identifiable information was collected. If a participant filled in and submitted the form, it was considered as an implied consent for his/her participation.

### Statistical analysis

Data was analyzed using the Statistical Package for the Social Sciences (SPSS) version 20.0 (SPSS, Chicago, IL, USA). Descriptive analysis was performed by mean, standard deviations for quantitative data and frequencies, percentages for qualitative data as applicable using frequency analysis for clinical features and epidemiological features. Logistic regression performed to predict potentially significant predictors of infection. P-value of ≤ 0.05 was considered statistically significant.

## Results

**Fig 1:**
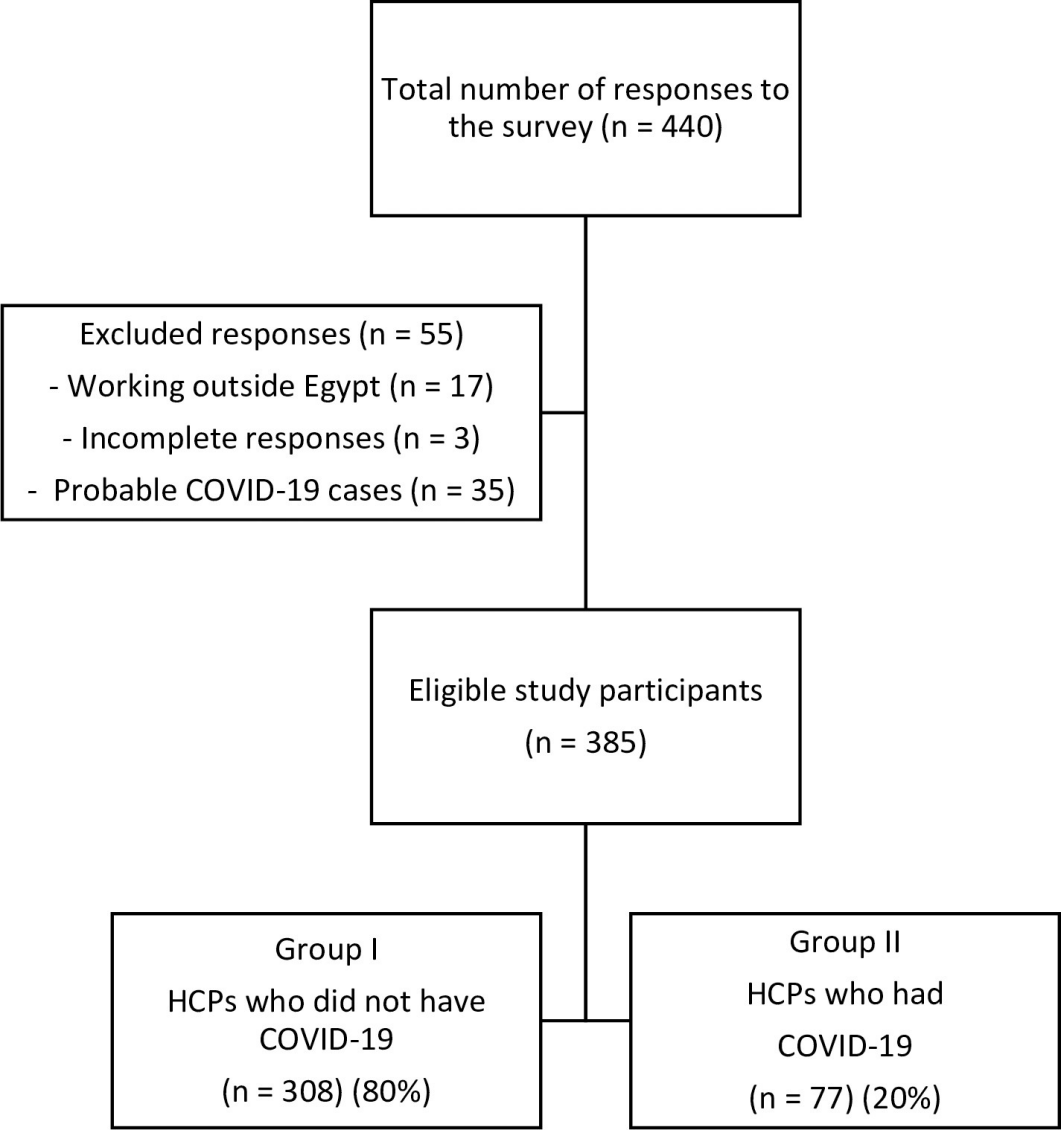
Flow chart of the study participants.

Out of 440 respondents to the online survey, a total of 385 HCPs were found eligible for inclusion in the study. The mean age of the included HCPs was 37.5±9.4 years. They were 215 (55.8%) males and 170 (44.2%) females. A flow chart of the study participants is demonstrated in Fig 1. Most of the study participants [308 (80%)] lay in group I (HCPs who did not have COVID-19) whereas participants in group II (HCPs who had COVID-19) were found to be 77 (20%).

Relevant epidemiological and clinical characteristics of group II participants are summarized in Table 1. They were 42 (54.5%) males and 35 (45.5%) females with mean age (39.09±10.43). Thirty-six (46.8%) of infected HCPs had associated co-morbidities. The majority of COVID-19 cases worked on full-time basis 72 (93.5%) and in hospital settings 71 (92.2%). Most of the cases have recovered from the disease (81.8%), with home isolation only (75.3%) and had mild COVID-19 symptoms (58.6%). Among the confirmed cases, 62.3% caught infection from symptomatic, and 37.7% from asymptomatic sources. Though 97.4% were on-duty, the exposure occurred during active healthcare provision in only 47 (61.1%) either from a COVID-19 patient or an infected colleague. When investigating the site exposure to COVID-19 patients inside the healthcare facility, 19.5% were exposed to COVID-19 patients inside the ICU. Some of the participants (29.9%) couldn’t precisely specify the exact site of exposure.

**Table 1:** Epidemiological and clinical characteristics of group II participants (HCPs who experienced COVID-19) (n = 77).

Variables	Number (%)
**Epidemiological characteristics**	
Age (years)	Mean ± SD (39.09±10.43)
Sex	
Male	42 (54.5)
Female	35 (45.5)
Timing of infection	
On duty	75 (97.4)
Not on duty	2 (2.6)
Source of SARS-CoV-2 infection	
From a COVID-19 household contact	4 (5.2)
From a COVID-19 patient at work	30 (39.0)
From an infected colleague (HCP)	17 (22.1)
Uncertain	26 (33.8)
Clinical status of source of infection	
Asymptomatic	29 (37.7)
Symptomatic	48 (62.3)
Site of exposure to COVID-19 patients	
Cleaning services	5 (6.5)
Emergency	5 (6.5)
Inpatient ward	5 (6.5)
Intensive care Unit	15 (19.5)
Laboratory	5 (6.5)
Outpatient area	4 (5.2)
Operating room	5 (6.5)
Pharmacy	2 (2.6)
Radiology/imaging	2 (2.6)
Reception area	2 (2.6)
Accommodation	1 (1.3)
Unknown	23 (29.9)
Others	4 (5.2)
**Clinical characteristics**	
COVID-19 symptoms	
Asymptomatic	7 (9.1)
Symptomatic	70 (90.9)
COVID-19 severity (n = 70)	
Mild	41 (58.6)
Moderate	21 (30.0)
Severe	8 (11.4)
Place of isolation	
At home	58 (75.3)
Admitted to hospital	19 (24.7)

Data are presented as mean ± SD for continuous variables and as number (percentage) for categorical variables.

COVID-19; Corona Virus Disease 2019, HCP; Healthcare Providers, SARS-CoV-2; Severe Acute Respiratory Syndrome Coronavirus 2.

Table 2 illustrates the comparison of personal and work-related characteristics among study participants. Male gender, physicians and nurses, working in hospital settings, and working on full time basis were more associated with COVID-19.

**Table 2:** Comparison between group I and group II as regards personal and work-related characteristics.

Variables	Group I 308 (80.0)	Group II 77 (20.0)	Total No (%)	*χ*2 Test	P value	OR (95% CI)
Age groups (years) (n = 385)						
≤ 29	54 (17.5)	13 (16.9)	67 (17.4)	0.21	0.21	1
30–39	164 (53.2)	31 (40.3)	195 (50.6)			0.79 (0.36–1.71)
40–49	60 (19.5)	21 (27.3)	81 (21.0)			1.45 (0.62–3.43)
≥ 50	30 (9.7)	12 (15.6)	42 (10.9)			1.66 (0.61–4.50)
X±SD	37.46±9.4					
Gender (n = 385)						
Male	128 (41.6)	42 (54.5)	170 (44.2)	4.2	0.04*	1
Female	180 (58.4)	35 (45.5)	215 (55.8)			0.59 (0.36–0.98)
Work experience (years) (n = 385)						
≤10	135 (43.8)	38 (49.4)	173 (44.9)	0.76	0.68	1
11–20	119 (38.6)	27 (35.1)	146 (37.9)			0.81 (0.45–1.45)
> 20	54 (17.5)	12 (15.6)	66 (17.1)			0.79 (0.36–1.71)
Occupation (n = 385)						
Physician	166 (53.9)	37 (48.1)	203 (52.7)	16.68^a^	0.003*	1
Pharmacist	41 (13.3)	4 (5.2)	45 (11.7)			0.44 (0.12–1.38)
Dentist	12 (3.9)	2 (2.6)	14 (3.6)			0.75 (0.11–3.76)
Nurse	55 (17.9)	20 (26.0)	75 (19.5)			1.63 (0.83–3.18)
Housekeeping	0 (0.0)	3 (3.9)	3 (0.8)			—
Technician	15 (4.9)	7 (9.1)	22 (5.7)			2.09 (0.7–5.81)
Others	19 (6.2)	4 (5.2)	23 (6.0)			0.94 (0.7–5.81)
Specialty^#^ (n = 300)						
Surgical specialties	60 (25.4)	10 (15.6)	70 (23.3)	7.25^a^	0.21	1
ICU & anesthesia	51 (21.6)	22 (34.4)	73 (24.3)			2.59 (1.09–6.51)
Medical specialties	75 (31.8)	18 (28.1)	93 (31)			1.44 (0.58–3.65)
Diagnostic specialties (laboratory and radiology)	43 (18.2)	9 (14.1)	52 (17.3)			1.26 (0.42–3.71)
Others	9 (3.8)	4 (6.3)	12 (4)			2.4 (0.51–100.81)
Place of work (n = 385)						
Hospital	242 (78.6)	71 (92.2)	313 (81.3)	7.53	0.006*	1
Non-hospital	66 (21.4)	6 (7.8)	72 (18.7)			0.31 (0.13–0.75)
Nature of work (n = 385)						
Full-time	254 (82.5)	72 (93.5)	326 (84.7)	5.79	0.02*	1
Part-time	54 (17.5)	5 (6.5)	59 (15.3)			0.327 (0.126–0.847)
Working hours/day (n = 385)						
<8	101 (32.8)	11 (4.3)	112 (29.1)	10.23	0.001*	1
≥ 8	207 (67.2)	66 (85.7)	273 (70.9)			2.93 (1.48–5.79)
Work in isolation hospital (n = 385)						
No	155 (50.3)	38 (49.4)	193 (50.1)	0.023	0.89	1
Yes	153 (49.7)	39 (50.6)	192 (49.9)			1.04 (0.63–1.71)
Working hours/day in isolation hospital (n = 192)						
<8	47 (30.9)	4 (10.0)	51 (26.5)	5.2	0.03*	1
≥ 8	106 (69.1)	35 (90.0)	141 (73.5)			3.2 (1.12–9.19)
Co-morbidities (n = 385)						
Absent	216 (70.1)	41 (53.2)	257 (66.8)	7.53	0.006*	1
Present	92 (29.9)	36 (46.8)	128 (33.2)			0.31 (0.129–0.745)

Group I: HCPs who did not have COVID-19, Group II: HCPs who had COVID-19.

* P ≤ 0.05 is significance.

^a^ Calculated using Fisher test.

^#^ Specialty for physicians, nurses and technicians,

CI; Confidence Interval, ICU; Intensive Care Unit, OR; Odds Ratio.

Working more than eight hours per day was associated with approximately three times higher risk of COVID-19 (OR: 2.9, 95%CI: 1.48–5.7, P < .0001). Similarly, for those working in isolation hospitals, working for more than eight hours per day posed them around three times higher risk for encountering the disease (OR: 3.2, 95%CI: 1.12–9.19, P = 0.03). Despite that specialties weren’t significantly associated with higher risk of COVID-19, yet working in intensive care units (ICU) and anesthesiology professions had around 3 times higher risk of getting the disease as compared to other specialties (OR: 2.59, 95%CI: 1.09–6.51, P = 0.21) (Table 2).

Statistically significant associations (Ps≤ 0.05) were observed between COVID-19 cases among HCPs and training on hand hygiene and adherence to IPC measures [(OR = 2.47, CI 1.21–5.03, P = 0.01), (OR = 2.37, CI 1.37–4.11, P = 0.002) respectively] (Table 3).

**Table 3:** Comparison between group I and group II as regards pertinent IPC training and compliance.

Variables	Group I 308 (80.0)	Group II 77 (20.0)	Total No (%)	*χ*2 Test	P Value	OR (95% CI)
Training on hand hygiene (n = 385)						
No	83 (26.9)	10 (13.0)	93 (24.2)	6.5	0.01*	1
Yes	225 (73.1)	67 (87.0)	292 (75.8)			2.47 (1.21–5.03)
Performing hand hygiene properly and according to WHO five moments (n = 385)						
No	41 (13.3)	16 (20.8)	57 (14.8)	2.7	0.09	1
Yes	267 (86.7)	61 (79.2)	328 (85.2)			0.59 (0.31–1.11)
Training on proper selection and use of PPE (n = 385)						
No	147 (47.7)	28 (36.4)	175 (45.5)	3.21	0.07	1
Yes	161 (52.3)	49 (63.6)	210 (54.5)			1.59 (0.95–2.68)
Using PPE in a proper way (n = 385)						
No	73 (23.7)	18 (23.4)	91 (23.6)	0.004	0.95	1
Yes	235 (76.3)	59 (76.6)	294 (76.4)			1.02 (0.75–1.83)
Adherence to IPC measures (n = 385)						
No	145 (47.1)	21 (27.3)	166 (43.1)	13.6	0.002*	1
Yes	163 (52.9)	56 (72.7)	219 (56.9)			2.372 (1.370–4.108)
Performing/assisting with/attending any AGP on a COVID-19 patient without wearing appropriate PPE (n = 259)						
No	163 (86.7)	59 (83.1)	222 (85.7)	0.55	0.46	1
Yes	25 (13.3)	12 (16.9)	37 (14.3)			1.32 (0.63–2.81)

Group I: HCPs who did not have COVID-19, Group II: HCPs who had COVID-19.

* P ≤ 0.05 is significance.

AGP; Aerosol Generating Procedure, CI; Confidence Interval, IPC: Infection prevention and control, OR; Odds Ratio, PPE: Personal Protective Equipment, WHO; World Health Organization.

**Fig 2:**
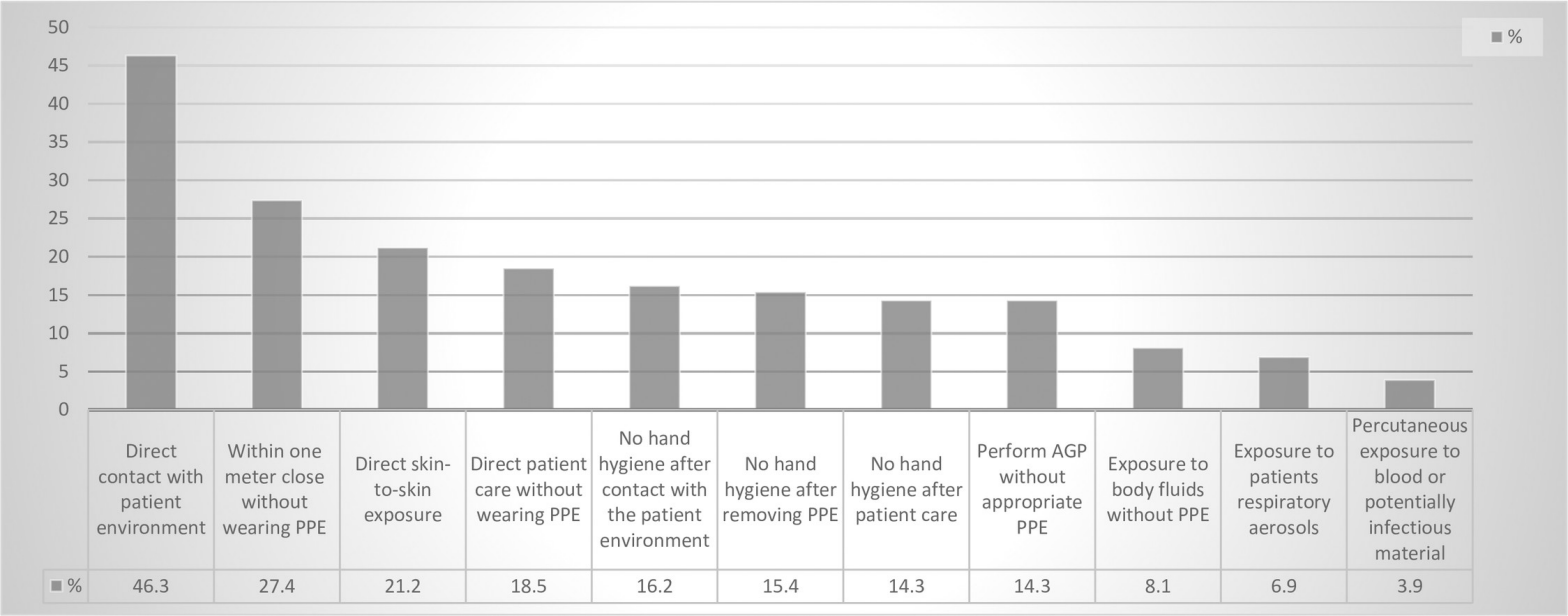
Frequencies of different exposures among HCPs who have dealt with a COVID-19 patient (n = 259).

Out of the 385 participants enrolled in the study, 259 of them showed a history of exposure to a COVID-19 case. Eighty-eight participants (34%) had a low exposure risk whereas 171 (66%) had a high exposure risk (OR = 0.78, CI 0.44–1.38, P = 0.39). Details of the risk exposure procedures are presented in Fig 2. Only 37 participants had either performed, assisted with or attended an aerosol generating procedure (AGP) performed to a COVID-19 patient without wearing appropriate personal protective equipment (PPE). The commonly reported procedures included using nebulizers (18.9%), tracheal intubation (17.8%), open air suctioning (13.9%), performing nasopharyngeal swab (8.1%), cardiopulmonary resuscitation (CPR) (7.7%), throat examination (4.6%), performing tracheotomy (0.8%) and bronchoscopy (0.4%).

Table 4 shows that the factors that significantly predict COVID-19 among respondents HCPs were the presence of co-morbidities (OR = 2.53, CI 1.47–4.38, P = 0.001), working more than eight hours per day in isolation hospital, (OR = 3.09, CI 1.02–9.35, P = 0.046), in addition to training on hand hygiene (OR = 2.31, CI 1.05–5.08, P = 0.038), and adherence to IPC measures (OR = 2.11, CI 1.16–3.81, P = 0.014).

**Table 4:** Logistic regression for factors predicting occurrence of COVID-19 among respondent HCPs.

Variables	B	Wald	P value	Exp(B)	95% C.I. for EXP(B)	
					Lower	Upper
Nature of work	-.673	1.665	.197	.510	.184	1.418
Work duration	.889	5.654	.017	2.433	1.169	5.065
Co morbidities	.931	11.104	.001*	2.536	1.467	4.384
Work duration in isolation hospitals	1.127	3.974	.046*	3.087	1.019	9.350
Occupation	-.014	.003	.960	.986	.569	1.709
Training on hand hygiene	.836	4.319	.038*	2.307	1.049	5.077
Adherence to IPC measures	.745	6.065	.014*	2.106	1.164	3.811
Constant	-7.519	19.869	.000	.001		

*P ≤ 0.05 is significant, B; unstandardized beta" regression coefficient", β;standardized beta, IPC; Infection prevention and control.

## Discussion

A serious task of healthcare institutions is to reduce the risk of occupationally acquired infections among HCPs. Perceiving the importance of such a task becomes more evident during the COVID-19 pandemic. Different factors have contributed to concerns for insufficiencies of measures for protection of HCPs’ health. Researchers from different countries tried to address the characteristics and factors related to COVID-19 among HCPs [25]. To the best of our knowledge, not enough data has been published about the clinical, epidemiological profile as well as the predicting factors related to the disease among HCPs in Egypt.

The study describes the working conditions for 385 HCPs in Egypt, along with factors related to COVID-19. They all reported the limited, inadequate supply and extended use of PPE in their institutions as the case in other countries [26–30]. A total of 77 (20%) HCPs with confirmed COVID-19 were described in the current study. Earlier reports revealed variable frequencies from different countries e.g., China (3.8%), Italy (11%), Spain (13.6%), and the United Kingdom (14%) [31]. The causes of such difference could not be accurately justified in the absence of national reports of infected cases among HCPs. Besides, the availability of PPE in resource limited settings, as is the case in many healthcare facilities in Egypt, may have contributed to the higher frequency of COVID-19 among HCPs in this study. Nevertheless, the high rates are alarming.

Most of the study participants reported a mild or moderate (83.2%) disease severity. Lai et al. [32] reported similar rates. This may be due to the relatively higher proportion of younger aged participants. Patients with severe and critical COVID-19 are usually older, with more frequent comorbidities [33]. In addition, mild degree can be easily detected by the HCPs with subsequent seeking for confirmatory investigation and early treatment [32]. The majority of the HCPs with COVID-19 (75.3%) were home isolated. Comparable results were recorded earlier (80–90%) [10]. This might show that treating mild patients outside a hospital setting with appropriate guidance from qualified medical professionals could be a reasonable approach when hospital capacities are limited [32]. Through governmental and voluntary initiatives, physicians have actually been providing free phone consultations and monitoring to mild cases isolated at home in Egypt.

Seven out of the 77 investigated HCPs (9.2%) were asymptomatic COVID-19 cases. Asymptomatic HCPs present a hidden infection source for silent infection spread among their patients, colleagues, and the community. This underscores the urgent need to implement additional control measures, e.g., implementing a national effective strategy for screening HCPs in contact with COVID-19 patients, prioritizing HCPs for testing. In addition to ensuring implementation of IPC strategies as a paramount measure to maintain safe environment for patients and HCPs [32].

As regards the source of infection, earlier WHO reports [34] suggested HCPs are being infected both in the workplace and in the community, most often through infected family members. Later reports from the CDC recorded household exposure in 27% of cases and healthcare exposure is reported in 55% of cases [9]. These could include prolonged or unprotected gatherings with colleagues or contact with contaminated surfaces. In this study, most of infected HCPs (61.1%) declared having contact with a COVID-19 patient, in healthcare settings either from a patient or a colleague HCP. Infection from a family member was also recorded (5.2%). Yet, the potential for exposure in multiple settings should be considered, especially as community transmission increases [9]. Added, transmission might come from unrecognized sources. In this report, 29 (37.7%) of infected HCPs stated the source of infections didn’t show any COVID-19 symptoms. As silent transmission of COVID-19 is a growing concern, the extent of pre-symptomatic and asymptomatic transmission should be considered very seriously. Previous studies reported that individuals may be most infectious during the pre-symptomatic phase of COVID-19 [35]. Moghadas et al., [36] recorded that pre-symptomatic and asymptomatic stages were responsible for more than 50% of the overall attack rate in COVID-19 outbreaks. Our findings highlight the serious need to reconsider changing the current Egyptian protocols for SARS-CoV-2 testing in healthcare settings and expand testing of suspected cases without symptoms as per the revised CDC guidelines [37]. The highest number of infected HCPs reported that the exposure occurred in ICU, this should be considered in providing special attention for preventive measures in ICU settings.

There is a growing interest in identifying risk factors of infection among HCPs. Policy makers and managers at healthcare facilities should consider the risk factors to adapt the protective measures in their context. Our study revealed long duty hours (more than 8 hours/day) and working on a full-time basis as a remarkable risk factor for COVID-19 infection. Ran et al., [38] and Mahngo et al., [39] reported similar findings. This, may be due to lack of rest, long-time exposure to infected patients [40], unprotected gathering with colleagues for food or chatting, and difficulty to tolerate wearing full PPE for a long period of time. Changes in the daily schedule to shorten the shift duration and decrease the daily working hours may be needed. In this report, males were at higher risk than female HCPs. In Egypt, most male HCPs work additional hours and in more than one healthcare facility, with a greater risk of exposure to infection. Among the infected cases, physicians and nurses were at higher risk of infection than other professions.

Current data shows the presence of Co-morbidity is a protective factor (OR 0.31) compared to absence of co-morbidity in acquiring COVID-19 infection. In earlier reports, comorbidities have been revealed as a major risk factor for encountering COVID-19 [41]. This may reflect a proper task allocations, and redistribution of the comorbid HCPs to jobs or health provision places with lower risk of exposure to infection during the pandemic [42].

Most importantly the inadequate training on hand hygiene and poor adherence to IPC were significant predictors of COVID-19. Though Egypt has a successful national IPC program for more than 20 years [43], yet there is still inadequate adherence to IPC and hand hygiene practices [44, 45]. Certain measures should take place on both national and facility levels to improve IPC practices, screen all HCPs for clinical signs of COVID-19 at the start of each shift to exclude HCP from work when ill, and avoid HCPs with comorbidities from working in high-risk areas. Strict implementation of IPC training with reinforcement of IPC teams should be a priority.

Our findings are in keeping with previous reports that after initiation of emergency responses to COVID-19, HCPs have not had enough time for systematic training and practice [8]. Professional supervision and guidance, as well as monitoring mechanisms, were lacking. Shortage in PPE aggravated the problem’s magnitude [8]. Hence, healthcare leaders should consider different means to conduct training on IPC measures, mainly hand hygiene and PPE use. On-line courses and mobile applications could be of help in this context. Reminders at workplace and remote monitoring by cameras could improve adherence to IPC practices, and combat future large-scale outbreak.

Limitations of the current study include the limited number of responders especially the infected HCPs. Severe cases were, by default, not included. Besides, since the study is a questionnaire-based study, so responses represent mostly subjective experience of the participants. Although, the online format limits the generalization of data, yet the study sheds some light on predicting factors that if considered could control the spread of COVID-19 among HCPs.

## Conclusion

This report identified factors related to COVID-19 among HCPs, with inferences to curb the infection spread among HCPs in Egypt, and probably other resource-limited countries. The 77 (20%) HCPs with confirmed COVID-19 included 7 asymptomatic cases (9.1%). Transmission from asymptomatic or presymptomatic sources accounted for 37.7% of cases. The presence of comorbidities, working more than 8 hours per day in isolation hospital, training on hand hygiene and adherence to IPC measures were significant predictors of COVID-19

Certain measures should be followed on both national and facility levels to improve IPC practices, screen all HCPs for clinical signs of COVID-19 at the start of each shift to exclude HCPs from work when ill, and exclude HCPs with comorbidities from working in high risk areas. Strict implementation of IPC training with reinforcement of IPC teams should be a priority. The working hours of HCPs in isolation hospitals should be reduced.

## Supporting information

S1 File (PDF)
