# Supplementary material for: Characteristics and predicting factors of Corona Virus Disease-2019 (COVID-19) among healthcare providers in a developing country
Source: PLoS One. 2021 Jan 20;16(1):e0245672. doi: 10.1371/journal.pone.0245672 (PMC7817002; doi:10.1371/journal.pone.0245672)
Supplement: S1 File — (PDF) [file pone.0245672.s001.pdf]

Questionnaire for Healthcare Providers about COVID-19 استبيان عن كوفيد-19 موجه لمقدمى الخدمة الصحية

This survey is directed to EGYPTIAN healthcare workers to explore the needed measures for better protection from COVID-19 spread among healthcare providers. Your accurate input will be of great help. Thank you for your time. This is designed for research purposes only by a group of experts and academic researchers from different Egyptian universities . Confidentiality of data is guaranteed. By submitting your response, you consented the participation in the study

شكرا لموافقتك على المشاركة في هذا الاستبيان الذي يهدف لمنع الاصابات بمرض الكوفيد 19 بين افراد الفريق الصحي وتحقيق حماية افضل لهم من العدوى اثناء العمل. تم اعداد الاستبيان بواسطة فريق من الخبراء والباحثين فى الجامعات المصرية المختلفة لغرض البحث العلمى فقط ولا يتم استخدام البيانات المذكورة فى اغراض اخرى. ملئ الاستبيان هو موافقة منك على المشاركة فى هذه الدراسة

* Required

1. Do you work in Egypt? هل تعمل داخل جمهورية مصر العربية؟ *

Mark only one oval.

- Yes نعم
- No لا

Personal and work related characteristics خصائص شخصية وخصائص طبيعة العمل

2. Age السن

____

3. Gender النوع *

Mark only one oval.

- Male ذكر
- Female انثي

4. For females healthcare workers فقط للزميلات

Mark only one oval per row.

	Yes نعم	No لا
Pregnancy هل يوجد حمل		
Lactation هل انت فى فترة الرضاعة		

5. Comorbid conditions - هل تعانى من اى من الامراض الاتية-اختر كل ما يناسب حالتك *

Check all that apply.

- [ ] ارتفاع ضغط الدم Hypertension
- [ ] مرض السكرى Diabetes mellitus
- [ ] امراض قلب مزمنة Heart disease
- [ ] امراض بالجهاز التنفسىLung diseases
- [ ] امراض الكلى Renal diseases
- [ ] امراض مناعة الذاتيةAutoimmune diseases
- [ ] On steroid therapy اتلقى علاج بالكورتيزون
- [ ] On immunosuppressive drugs اتلقى علاج مثبط للمناعة
- [ ] Immunosuppressive disorders امراض نقص/خلل الجهاز المناعى
- [ ] لايوجدNone
- [ ] Other اخرى

6. Other co-comorbidity فى حالة وجود امراض اخرى لم تذكر فى القائمة السابقة ...من فضلك أذكرها

7. Governorate المحافظة *

Mark only one oval.

- Alexandria Governorate الاسكندرية
- Aswan Governorate اسوان
- Asyut Governorate اسيوط
- Beheira Governorate البحيرة
- Beni Suef Governorate بني سويف
- Cairo Governorate القاهرة
- Dakahlia Governorate الدقهلية
- Damietta Governorate دمياط
- Faiyum Governorate الفيوم
- Gharbia Governorate الغربية
- Giza Governorate الجيزة
- Ismailia Governorate الاسماعيلية
- Kafr El Sheikh Governorate كفر الشيخ
- Luxor Governorate الاقصر
- Matruh Governorateمطروح
- Minya Governorate المنيا
- Monufia Governorate المنوفية
- New Valley Governorate الوادي الجديد
- North Sinai Governorate جنوب سيناء
- Port Said Governorate بور سعيد
- Qalyubia Governorate القليوبية
- Qena Governorate قنا
- Red Sea Governorate البحرالاحمر
- Sharqia Governorate الشرقية
- Sohag Governorate سوهاج
- South Sinai Governorate شمال سيناء
- Suez Governorate السويس

8. Work experience: عدد سنوات الخبرة *

Mark only one oval.

- < 5 years أقل من خمس سنوات
- 6-10 years
- 11- 15 years
- 16-20 years
- > 20 years أكثر من 20 سنة

9. Job الوظيفة *

Mark only one oval.

- Physician طبيب
- Pharmacist صيدلى
- Dentist طبيب اسنان
- Physiotherapist اخصائى علاج طبيعى
- Nurse تمريض
- Technician كميائي /فني
- Worker عامل
- Others تخصصات اخرى

10. Specialty التخصص (للاطباء البشريين والفنيين والعمال)

11. Other job فى حالة وجود وظيفة اخرى لم تذكر فى القائمة السابقة ...من فضلك أذكرها

12. Your Healthcare facility type (select primary location) حدد مكان عملك الاصلى *

Mark only one oval.

- Hospital مستشفى
- Primary health center مركز رعاية صحية اولية
- Outpatient clinic عيادة خارجية
- Rehabilitation center مركز تأهيل
- Quarantine الحجر الصحى
- Oncology centre مركز اورام
- Other:

13. Other workplace فى حالة وجود مكان عمل آخر لم يذكر فى القائمة السابقة ...من فضلك أذكره

14. select what suits you ما طبيعة مدة عملك *

Mark only one oval.

- Full time وقت كامل
- Half time نصف الوقت

15. Working hours/day ما عدد ساعات العمل فى اليوم الواحد *

Mark only one oval.

- less than 8 hours اقل من 8 ساعات
- 8 hours مدة العمل 8 ساعات
- more than 8 hours اكتر من 8 ساعات

16. Have you worked in a COVID-19 isolation Hospital or a healthcare receiving suspected COVID-19 patients? هل عملت فى اى من مستشفيات العزل الخاصة بالكوفيد-19 او بمستشفى تستقبل حالات اشتباه كوفيد-19 *

Mark only one oval.

- yes نعم
- no لا

17. if yes, mention the duration (days) ما عدد ايام عملك بمستشفى العزل

____

18. Working hours/day in COVID-19 isolation hospital ما عدد ساعات العمل فى مستشفى عزل كوفيد-19 فى اليوم الواحد

Mark only one oval.

- less than 8 hours اقل من 8 ساعات
- 8 hours مدة العمل 8 ساعات
- more than 8 hours اكتر من 8 ساعات

Infection control measures during COVID-19 pandemic اجراءات الوقاية من العدوى اثناء جائحة الكوفيد-19

19. What are the most successful infection control measures against COVID-19 at your workplace? (Check all that apply). ماالكثر اجراءات مكافحة العدوى نجاحا داخل مكان عملك - اختركل ما تراه مناسبا من الاجراءات التالية *

Check all that apply.

- [ ] Policies & procedures وجود سياسات وإجراءات واضحة لمكافحة العدوي
- [ ] Active infection control team فريق مكافحة عدوي نشط
- [ ] Triage فرز المرضي
- [ ] Restricted area for COVID-19 patients منطقة محددة لعزل مرضى الكوفيد 19
- [ ] Patients adhere to wearing masks يلتزم المرضى بارتداء أقنعة
- [ ] Availability of PPE supplies توافر معدات الوقاية الشخصية
- [ ] Good quality PPE معدات الوقاية الشخصية ذات نوعية جيدة
- [ ] Availability of hand hygiene supplies توافر مستلزمات نظافة اليدين
- [ ] Well-equipped isolation wards/rooms عنابر/غرف عزل مجهزة تجهيزًا جيدًا
- [ ] Infection control training program برامج التدريب علي اجراءات مكافحة العدوي
- [ ] Negative pressure room for aerosol generating procedures غرفة ضغط سلبية للاجراءات المنتجة للرذاذ
- [ ] None لا يوجد
- [ ] Other اجراءات أخرى

20. Other infection control measures فى حالة وجود اجراءات أخرى لم تذكر فى القائمة السابقة ...من فضلك أذكرها

____

____

____

____

____

21. اختر ما يتلائم معكٍ Select the suitable for you *

Mark only one oval per row.

	نعم Yes	لا No
Were you trained to properly apply hand hygiene?هل تم تدريبك على تطبيق نظافة اليدين بشكل صحيح؟	◯	◯
Do you apply hand hygiene in a proper way and according to WHO 5 moments هل تلتزم بتطهير الأيدى بطريقة سليمة تبعا لمنظمة الصحة العالمية وباتباع الخمس لحظات الهامة	◯	◯
Were you trained to properly select and use PPEهل تم تدريبك على اختيار معدات الوقاية الشخصية واستخدامها بشكل صحيح؟	◯	◯
Do you use PPE in a proper wayهل تستخدم معدات الوقاية الشخصية بطريقة مناسبة	◯	◯

22. Who observes you while performing hand hygiene, donning and doffing PPE - check all that apply من كان يتابع استخدامك لتطهير الايدى و استخدام الواقيات الشخصية- اختر كل ما يناسبك

Check all that apply.

- [ ] Infection control officer مسئول مكافحة العدوى
- [ ] Quality officer مسئول الجودة
- [ ] A colleague احد الزملاء
- [ ] A senior/supervisor زميل اكبر منى او رئيسى فى العمل
- [ ] Myself انا
- [ ] None لا يتابعنى احد

23. How often do you use single use gloves for one patient ما معدل استخدامك القفاز ذو الاستخدام الواحد لمريض واحد *

Mark only one oval.

- ( ) Always, as recommended دائمًا على النحو الموصى به
- ( ) Mostly معظم الوقت
- ( ) Occasionally من حين لآخر
- ( ) Rarely نادرًا

24. How often do you use medical/ surgical mask ما معدل استخدامك القناع الطبي / الجراحي *

Mark only one oval.

- ( ) Always, as recommended دائمًا على النحو الموصى به
- ( ) Mostly معظم الوقت
- ( ) Occasionally من حين لآخر
- ( ) Rarely نادرًا

25. How often do you use face shield or goggles/protective glasses ما معدل استخدامك واقى الوجه / نظارات واقية *

Mark only one oval.

- ( ) Always, as recommended دائمًا على النحو الموصى به
- ( ) Mostly معظم الوقت
- ( ) Occasionally من حين لآخر
- ( ) Rarely نادرًا

26. Did you experience any shortage in PPE supplies during the pandemic? هل هناك نقص فى الاردية الواقية *

Mark only one oval.

- ( ) Yes نعم
- ( ) No لا

27. If yes, which type (فى حالة وجود نقص فى كميات الأردية الواقية..من فضلك حدد النوع) يمكنك اختيار اكثر من صنف

Check all that apply.

- [ ] Gloves القفازات ذات الاستخدام الواحد
- [ ] Gowns الجاونات
- [ ] N95 respirator ماسك N95
- [ ] Surgical masks الماسك الجراحى
- [ ] Googles/face shield نظارات/واقى الوجه

28. Did you have to extend the use of PPE because of its shortage? هل استخدمت الاردية الواقية لمدة اطول من المعتاد بسبب نقص هذة الاردية *

Mark only one oval.

- ( ) Yes نعم
- ( ) No لا

29. If yes, which type (اذا كنت استخدمت الاردية الواقية لمدة اطول من المعتاد..من فضلك حدد النوع) يمكنك اختيار اكثر من صنف

Check all that apply.

- [ ] Gloves القفازات ذات الاستخدام الواحد
- [ ] Disposable gowns الجاونات احادية الاستخدام
- [ ] N95 respirator N95 ماسك
- [ ] Surgical masks الماسك الجراحى
- [ ] Googles/face shield نظارات/واقى الوجه

30. How often do you use disposable gown ما معدل استخدامك للجاون احادي الاستخدام *

Mark only one oval.

- ( ) Always, for one patient دائما لمريض واحد
- ( ) For 12 hours لمدة 12 ساعة
- ( ) For 24 hours لمدة 24 ساعة
- ( ) For 1-3 days لمدة 1-3 أيام
- ( ) Rarely نادرًا

31. How often do you use Coverall ما معدل استخدامك بدلة واقية كاملة *

Mark only one oval.

- ( ) Always, for one patient دائما لمريض واحد
- ( ) For 12 hours لمدة 12 ساعة
- ( ) For 1-3 days لمدة 1-3 أيام
- ( ) Rarely نادرًا

32. Do you re-sterilize N95 respirator هل تعيد تعقيم ماسكات الN95 *

Mark only one oval.

- Yes نعم
- No لا

Dealing with Covid-19 patient 19 التعامل مع مريض مصاب بالكوفيد

33. Have you dealt with a COVID-19 patient? هل تعاملت مع مريض مصاب بالكوفيد؟ *

Mark only one oval.

- Yes نعم *Skip to question 34*
- No لا *Skip to question 37*

Untitled Section

34. Select what matches you in the following situations اختر ما حدث معك فى كل موقف من المواقف التالية *

Mark only one oval per row.

	yes نعم	no لا
1- Was there a direct skin-to-skin exposure to a COVID-19 patient? عندما تعاملت مع الحاله هل حدث تلامس مباشر بين جلدك وجلد المريض	◯	◯
2- Were you within one meter of a COVID-19 patient while not wearing appropriate PPE or had issues with your PPE (e.g., tears, removed while in patient area)?هل كنت موجود علي مسافة متر من المصاب دون ان ترتدي اي واقيات شخصية او ارتديتها وحدث بها ثقب او تلف او تم خلعها وانت داخل منطقة رعاية المريض	◯	◯
3- Did you have a direct contact with environment (e.g., bed, linens, medical equipment, frequently touched surfaces, bathroom) where the patient received care هل تعاملت تعامل مباشر مع البيئة المحيطة بالمريض مثل (الملايات- الحمامات- الاجهزة الطبية)	◯	◯
4- Did you provide direct care to a confirmed COVID-19 patient while not wearing appropriate personal protective equipment (PPE) or had issues with your PPE (e.g., tears, removed while in patient area)?هل قدمت خدمة طبية مباشرة للمصاب دون ان ترتدي اي واقيات شخصية او ارتديتها وحدث بها ثقب او تلف او تم خلعها وانت داخل منطقة رعاية المريض	◯	◯
6- Did you fail to perform hand hygiene after providing a direct patient care هل فشلت فى القيام بغسيل الايدي الصحي بعد تقديم الرعاية للمريض	◯	◯
6- Did you fail to perform hand hygiene after having direct contact with the environment where a COVID-19 patient received care هل فشلت فى القيام بغسيل الايدي الصحي بعد لمس البيئة المحيطة بالمريض	◯	◯
7- Did you fail to perform hand hygiene after removing your PPE هل فشلت فى القيام بغسيل الايدي الصحي بعد خلع الواقيات الشخصية	◯	◯
8- Did you perform or assist or attend with any aerosol-generating procedure (AGP) on a COVID-19 patient, without wearing appropriate personal protective equipment (PPE)? Or had issues with my PPE (e.g., torn PPE or removed while in patient area)? هل قمت بعمل او بالمساعدة او الحضور اثناء اجراء للمصاب كان به تناثر للرذاذ دون ارتداء الواقايات الشخصية او كانت ممزقة او تم خلعها داخل منطقة رعاية المريض	◯	◯
9- Did you handle body fluid or other specimens from the patient without wearing appropriate PPE? Or had issues withyour PP E (e.g., torn PPE or removed while in patient area)? هل تعاملت مع افرازات او عينات المريض دون ارتداء الواقايات الشخصية او كانت ممزقة او تم خلعها داخل منطقة رعاية المريض	◯	◯
10- Did you have direct exposure (to your skin or mucous membrane) to a COVID-19 patient's respiratory secretions or bodily	◯	◯

fluid? هل تعاملت مع الحاله وحدث تلامس مباشر بين جلدك او اغشيتك المخاطية مع اى من سوائل الجسم او الافرازات التنفسية للمريض		
11- Did you have a percutaneous exposure (e.g., needle stick, cut, puncture) with material potentially contaminated with body fluid, blood, or respiratory secretions.هل تعاملت مع الحاله وحدث اختراق او قطع لجلدك مع تلامس لمادة ملوثة بدم او سوائل الجسم او الافرازات التنفسية للمريض	◯	◯

35. If you attend/perform any aerosol generating procedures, what type is it? اذا كنت قمت باجراء او تواجدت اثناء اجراء احد الاجراءات المنتجة للرذاذ ؟ حدد هذا الاجراء من القائمة التالية وفى حالة وجود اكثر من اجراء اذكرهم جميعا

Check all that apply.

- [ ] Tracheal intubation تركيب انبوبة حنجرية
- [ ] Nebulizer treatment (نيوبيلايزر) علاج بالاستنشاق)
- [ ] Open airway suctioning تشفيط
- [ ] Collection of sputum تجميع بصاق
- [ ] Tracheotomy شق حنجري
- [ ] Bronchoscopy ميكروسكوب شعبي
- [ ] (Cardiopulmonary resuscitation (CPR انعاش قلبي رئوي
- [ ] Throat examination in pediatrics فحص الحلق فى خالات الاطفال
- [ ] Nasopharyngeal swab collection سحب مسحة من الحلق
- [ ] Others اجراءات اخرى

36. Other aerosol generating procedures فى حالة وجود اجراءات أخرى لم تذكر فى القائمة السابقة ...من فضلك أذكرها

Untitled Section

37. Did you get infected with COVID-19? هل تعرضت للاصابة بمرض الكوفيد-19 *

Mark only one oval.

◯ Yes نعم

◯ No لا

COVID-19 infection خصائص الاصابة بمرض الكوفيد19

38. When did you get infected متى تعرضت للاصابة؟ *

Mark only one oval.

◯ وأنا على قوة العمل I was on duty

◯ لم اكن على قوة العمل I was not on duty

39. What was the source of your COVID-19 infection من هو مصدر العدوي *

Mark only one oval.

- From a COVID-19 household contact احد المخالطين بالمنزل كان مصابا بالكوفيد
- From a COVID-19 patient at work مريض كوفيد في مكان العمل
- From a colleague من احد الزملاء فى مكان العمل
- Uncertain لا استطيع تحديده
- Others اخرون

40. Was the source of infection symptomatic? هل كان مصدر العدوي يشكو من اي اعراض؟

Mark only one oval.

- Yes نعم
- No لا

41. Where did the exposure to COVID-19 patients occur? - check all that apply. - حدد مكان اصابتك بالعدوي داخل مكان عملك اختر كل ما تراه مناسب من الأماكن التالية *

Check all that apply.

- Cleaning services خدمات النظافة
- Emergency room غرفة الطوارئ
- Inpatient ward الاقسام الداخلية
- Intensive care Unit وحدة الرعاية المركزة
- Laboratory المعمل
- Outpatient area العيادات الخارجية
- Operating room غرفة العمليات
- Pharmacy صيدلية
- Radiology/imaging الاشعه
- Reception area الاستقبال
- Transport اثناء نقل المريض
- Triage مكان الفرز
- Administrative offices مكاتب ادارية
- On call rooms السكن
- Unknown لا اعلم
- Other أخرى

42. Other site فى حالة وجود مكان اصابة آخر لم يذكر فى القائمة السابقة ...من فضلك أذكره

43. How were you diagnosed as COVID-19 positive (Select all that applies) كيف تم تشخيص اصابتك بمرض الكوفيد -19- اختر كل ما تراه مناسب فى طرق التشخيص التالية *

Check all that apply.

- Clinical بالفحص الاكلينيكي
- CT chest بالاشعة المقطعية
- Serology تحليل الدم السيروولوجى
- PCR اختبار البى سى ار

44. Your COVID-19 symptoms ما هي اعراض الكوفيد-19 التي ظهرت عليك *

Mark only one oval.

- Asymptomatic لم تظهر اعراض
- Mild أعراض بسيطة
- Moderate اعراض متوسطة
- Severe اعراض شديدة

45. Where have you been isolated ماهو مكان العزل *

Mark only one oval.

- At home عزل منزلى
- Admitted to hospital داخل مستشفي
- Admitted to a COVID-19 isolation hospital داخل مستشفي عزل

46. Your clinical outcome ماهو وضع الحالة اكلينيكيا *

Mark only one oval.

- Recoverd تماثلت للشفاء
- Complicated حدوث مضاعفات
- Under treatment مازلت تحت العلاج

This content is neither created nor endorsed by Google.

Google Forms
